# Bioenergetic modulation with the mitochondria uncouplers SR4 and niclosamide prevents proliferation and growth of treatment-naïve and vemurafenib-resistant melanomas

**DOI:** 10.18632/oncotarget.26421

**Published:** 2018-12-11

**Authors:** James L. Figarola, Jyotsana Singhal, Sharad Singhal, Jyotirmoy Kusari, Arthur Riggs

**Affiliations:** ^1^ Division of Diabetes and Metabolic Diseases Research, Beckman Research Institute, City of Hope National Medical Center, Duarte, CA 91010, USA; ^2^ Department of Medical Oncology, Beckman Research Institute, City of Hope National Medical Center, Duarte, CA 91010, USA

**Keywords:** BRAF, MAPK, mitochondria, uncoupler, xenograft

## Abstract

BRAF mutations are detected in >50% of all melanomas. These mutations impair the LKB1-AMPK signaling, an important metabolic pathway associated with cell growth, proliferation and survival. Melanoma patients with BRAF mutations are usually treated with BRAF inhibitors such as vemurafenib, but responses are short-lived as drug resistant tumors metabolically switch to mitochondrial oxidative phosphorylation (OXPHOS) to escape metabolic stress-induced BRAF inhibition. Additionally, a large subset of melanoma utilizes OXPHOS in their metabolism, which can confer *de novo* resistance to BRAF inhibitors. Therefore, uncoupling of OXPHOS to perturb energy homeostasis and to indirectly stimulate AMPK could be a novel treatment for melanoma and to overcome intrinsic and acquired resistance to BRAF inhibitors. Here, we investigated the effects of SR4 and niclosamide, two small molecule mitochondria uncouplers, on the growth and proliferation of treatment-naïve and vemurafenib-resistant melanomas *in vitro* and *in vivo*. SR4 and niclosamide inhibited melanoma proliferation irrespective of BRAF/NRAS status. Melanomas with greater OXPHOS phenotype (higher OCR/ECAR), with LKB1 mutation, or with acquired resistance to vemurafenib displayed greater sensitivity to both uncouplers. More importantly, SR4 and niclosamide inhibited tumor growth in both treatment-naïve and vemurafenib-resistant xenograft mice models. Mechanistic studies indicate both uncouplers induced energetic stress, modulated the AMPK-mTOR pathway, and promoted apoptosis without affecting MEK-ERK MAPK signaling. These results suggest that uncouplers such as SR4 and niclosamide may be useful as first line treatment against melanoma regardless of BRAF/NRAS status, and as an adjuvant therapy for patients failing MAPK inhibitors.

## INTRODUCTION

Melanoma is the deadliest form of skin cancer and its incidence continues to increase worldwide. In the United States, the estimated number of yearly cases and deaths in 2018 were 91,270 and 9,320, respectively [1]. Approximately 50-60% of melanomas have a mutation in the BRAF (v-Raf murine sarcoma viral oncogene homolog B protein) kinase. All the mutations occur within the kinase domain, and the specific V600E missense valine to glutamic acid mutation accounts for approximately 80–90% of BRAF mutations [2, 3]. This mutation leads to a conformational change resulting in constitutive activation of BRAF, and consequently of the MEK/ERK MAPK pathway, promoting survival and proliferation of melanoma cells [4, 5]. Currently, there are two BRAF mutation inhibitors (vemurafenib and dabrafenib) that are approved by the U.S. FDA to treat stage 3 or 4 melanoma with positive BRAF^V600E^ or BRAF^V600K^ mutation. However, durable responses to BRAF mutation inhibitors are rare and most patients invariably relapse with drug-resistant disease within 6-8 months [6]. Mechanisms of acquired resistance to BRAF inhibition can be subdivided in two groups: MAPK-dependent and MAPK-independent. The former is primarily due to MEK/ERK reactivation resulting from amplification of BRAF, BRAF splicing, NRAS mutation, MEK mutation and loss of NF1, while the latter resistance mechanisms include up-regulated receptor tyrosine kinases (RTKS), activating mutations in AKT and loss of function mutations in PTEN and overexpression of COT [7–8]. Combination with BRAF inhibitors and FDA-approved MEK inhibitors (trametinib and cobimetinib) significantly improves the progression free survival but patients also eventually relapse due to drug resistance [9] and the percentage of patients with adverse events is higher in this combination regimen than BRAF inhibition monotherapy [10]. Studies also showed that acquired resistance to BRAF inhibition can confer cross-resistance to combined BRAF/MEK inhibition [11]. Thus, despite the recent success in the development of targeted therapies for melanoma, the problem of drug resistance and the rapidly rising incidence and morbidity rate of melanoma underscore the urgency to better understand its pathogenesis and identify potential therapeutic targets and treatment strategies.

The AMP-activated serine/threonine protein kinase (AMPK) and its upstream kinase, LKB1, act to both monitor and restore cellular energy in response to energy depletion [12]. The LKB1 tumor suppressor phosphorylates and activates AMPK when cellular energy levels are low, thereby suppressing growth through multiple pathways, including inhibiting mTORC1 (mammalian target of rapamycin complex 1) kinase that is activated in the many human cancers [13]. Studies suggested that many melanomas have low AMPK and high mTOR activity due to mutations that enable them to escape energetic stress and continue proliferation [14, 15]. The BRAF oncogene has recently been implicated in cellular metabolism in melanoma, specifically in mediating resistance to energetic stress. BRAF^V600E^ mutation has been shown to turn off the LKB1-AMPK pathway by phosphorylating LKB1, preventing its ability to bind and activate AMPK [14, 16]. Moreover, BRAF affects oxidative metabolism through microphthalmia-associated transcription factor (MITF)-dependent control of peroxisome proliferator-activated receptor gamma coactivator 1-alpha (PGC1α), the master regulator of mitochondria biogenesis [17]. Consequently, melanomas resistant to BRAF/MEK inhibitors have increased mitochondria biogenesis and metabolically switch to oxidative phosphorylation (OXPHOS) through upregulation of MITF and PGC1α [17–20]. In addition, several metabolic profiling and flux analyses studies demonstrated that OXPHOS complexes are functional in two-thirds of primary human melanoma tissues and majority of metastatic melanoma derived a large fraction of energy from OXPHOS, even under hypoxia [21–24]. One study even showed that freshly isolated patient derived stage IV metastatic melanoma exhibited substantially higher rates of OXPHOS than human melanocytes [23]. Considering that a large number of metastatic melanomas are actively using OXPHOS, while others could adapt to MAPK inhibitors by driving oxidative metabolism through MITF-PGC1α, suggest that targeting mitochondrial OXPHOS and/or activating AMPK could be an effective therapeutic approach to inhibit melanoma growth and proliferation.

**Figure 1:**
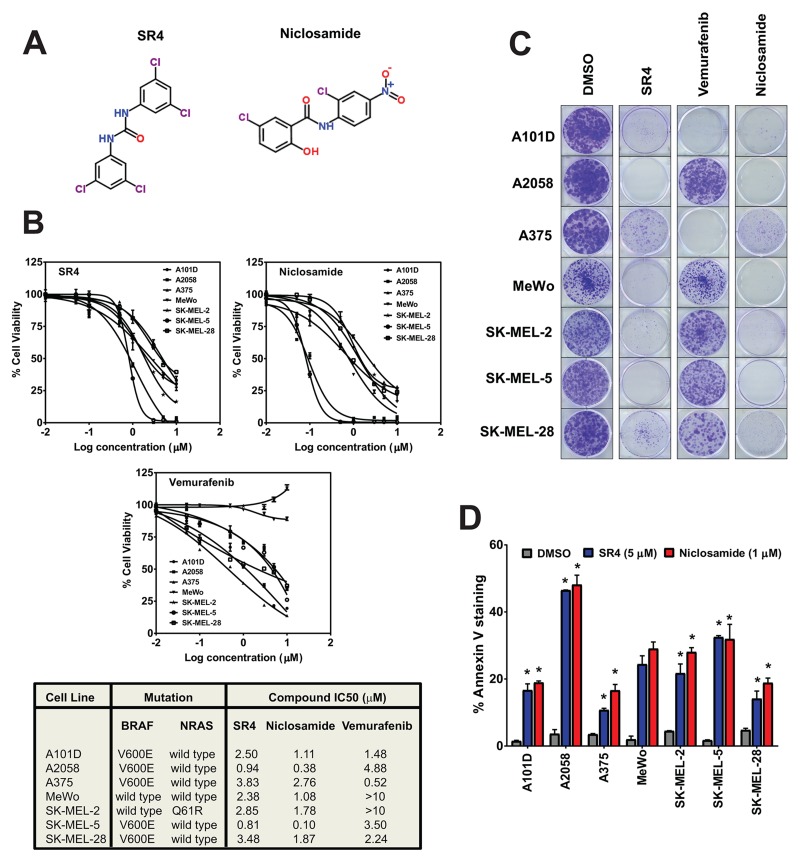
SR4 and niclosamide inhibit proliferation of melanoma irrespective of BRAF/NRAS status **(A)** Chemical structures of SR4 and niclosamide. **(B)** Dose response curves and IC_50_ values of the seven melanoma lines treated with SR4, niclosamide and vemurafenib. Cell viability was measured by Cell Titer Glo assay after 48 h. IC_50_ values were calculated using GraphPad prism (*n* = 3). **(C)** Representative colony formation assays of melanoma cells following treatments with DMSO control or 1 μM each of SR4, niclosamide and vemurafenib. **(D)** Annexin V staining after treatment with SR4 (5 μM) and niclosamide (1 μM) for 48 h (mean ± SEM, n = 3). ^*^P < 0.05 vs. DMSO control.

In this study, we investigated the metabolic effects of SR4 and niclosamide (Figure 1A), two small molecules that were recently identified as mitochondria uncouplers, against treatment naïve wild type, BRAF^V600E^ and NRAS mutant, and BRAF inhibitor (vemurafenib)-resistant melanomas. Mitochondria uncouplers exert their effects by dissipating the proton gradient formed by the electron transport chain, thus uncoupling ATP production and causing energetic stress. We have shown previously that SR4 is a bonafide uncoupler that prevented cell proliferation and promoted cell death in many human cancers including leukemia, lung cancer, melanoma, and hepatocarcinoma *in vitro* and in animal models [25–28]. Similarly, niclosamide is an FDA approved antihelminth drug for the past 50 years, and recent studies have demonstrated its uncoupling and anticancer activities against a variety of human cancers *in vitro* and *in vivo* [29–34]. However, the effects of both compounds in melanoma with various oncogenic driver mutations and with drug-resistant melanoma have not been investigated, as well as the metabolic signaling mechanisms of both uncouplers in melanoma. Our current data showed that the anti-proliferative and anti-tumor effects of both SR4 and niclosamide *in vitro* and in mice xenograft studies result from uncoupling of mitochondrial OXPHOS that induces energetic stress on cells, consequently leading to AMPK activation and mTOR inhibition without any effects on ERK/MEK MAPK signaling. More importantly, both uncouplers were more potent to BRAF-inhibitor resistant melanoma as a consequence of drug-induced metabolic switch to OXPHOS phenotype.

## RESULTS

### SR4 and niclosamide inhibit melanoma cell proliferation *in vitro* independent of BRAF/RAS mutations

The anti-proliferative effects of both SR4 and niclosamide were evaluated in melanoma cells with wild type *BRAF* (Mewo, SK-MEL-2), *BRAF*^V600E^ (A101D, A375, A2058, SK-MEL5, SK-MEL-28) and *NRAS* (SK-MEL-2) mutations using the Cell Titer Glo cell viability assay. Treatment with either SR4 or niclosamide for 48 h inhibited proliferation of all melanoma cells, with IC_50_ values of 0.81- 3.83 μM and 0.10-2.76 μM for SR4 and niclosamide, respectively, with the latter being more potent across all seven melanoma cell lines tested (Figure 1B). There was no correlation between the responses to SR4 or niclosamide and the BRAF/NRAS mutation status of each cell line, but both A2058 and SK-MEL-5, known LKB1 mutant [35] and LKB1 null cells [36], respectively, were the most sensitive to both uncouplers. As expected, vemurafenib had no anti-proliferative effects on BRAF wild type cells MeWo and SK-MEL-2; instead it promoted increased cell proliferation in the latter. All three compounds have little or no toxicity to normal human melanocytes (IC_50_ not detectable at 10 μM, data not shown). We also investigated the long-term anti-proliferative effects of SR4 and niclosamide in comparison with vemurafenib using colony formation assay. Even at 1 μM concentration, both SR4 and niclosamide almost completely inhibited colony formation of all seven melanoma cells after 10 days incubation with the compounds, whereas vemurafenib displayed variable effects on BRAF^V600E^ mutants and failed to inhibit colony growth of wild type BRAF cells (Figure 1C). To further characterize the anti-proliferative effects of both SR4 and niclosamide, we treated the cells with the compounds for 48 h and then measured apoptosis by Annexin V-PI staining and flow cytometry. Both uncouplers promoted apoptosis in all melanoma cells (Figure 1D). Consistent with results obtained from the cell viability test, SK-MEL-5 and A2058 showed the highest apoptotic rates among these cell lines when treated with either compound. These data suggest that cells that are LKB1 deficient are most susceptible to SR4 and niclosamide.

### Metabolic phenotype correlates with susceptibility to SR4 and niclosamide

**Figure 2:**
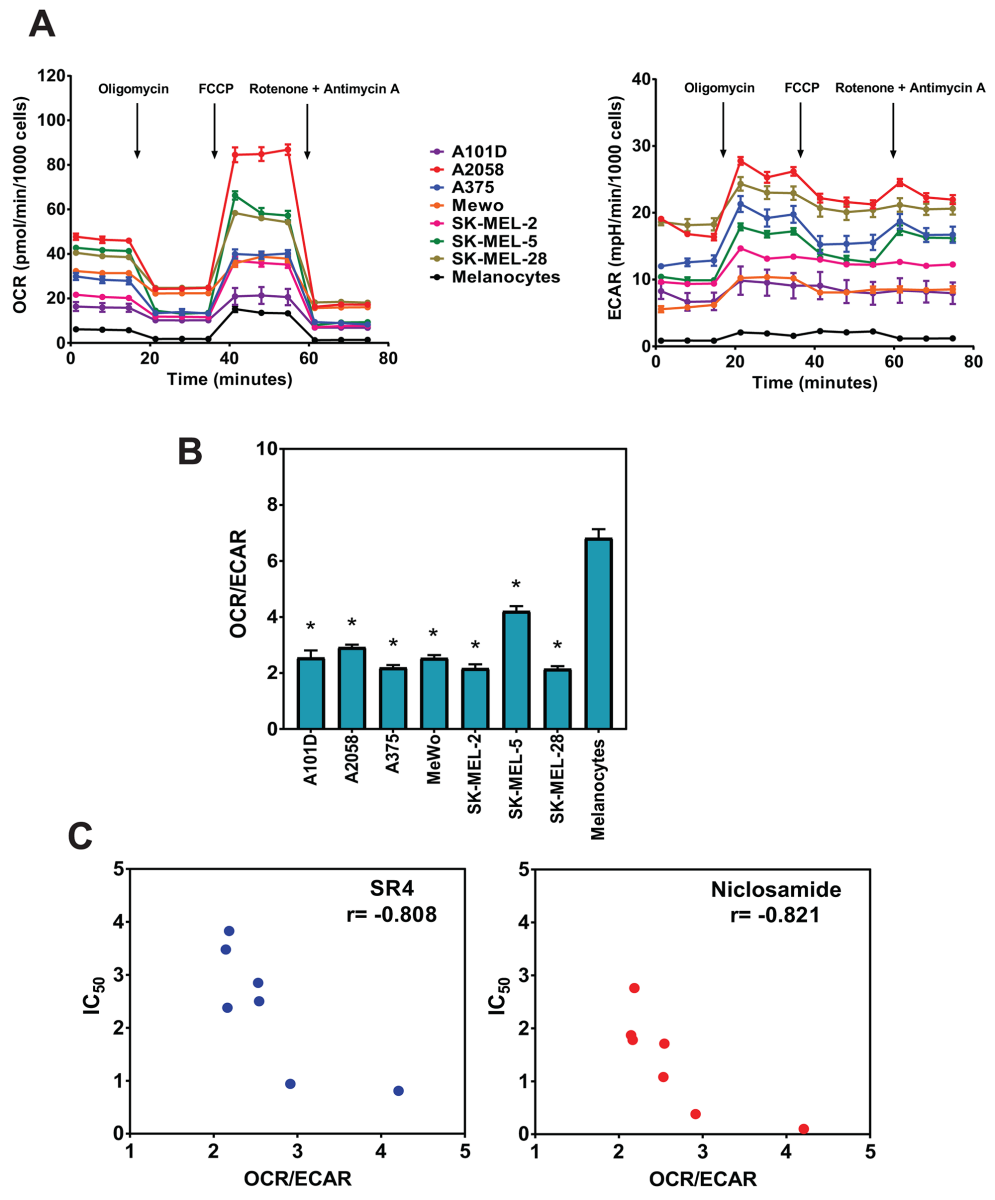
Metabolic profiling of melanoma cells and primary melanocytes **(A)** OCR and ECAR measurements of each cell line using the Seahorse XF96 flux analyzer after successive addition of oligomycin (1 μM), FCCP (1 μM), rotenone/antimycin A (1 μM/1 μM). **(B)** OCR/ECAR ratios for melanoma cells and primary melanocytes. Data are means from 6-8 wells ± SEM, and are representative rates of two independent experiments. ^*^P < 0.05 vs. primary melanocytes. **(C)** Correlation of baseline OCR/ECAR with IC_50_ values for SR4 and niclosamide (from Figure 1B). Pearson correlation coefficient (r) was calculated using GraphPad Prism.

Given the variable response of each of the melanoma cells to both SR4 and niclosamide, we next examined whether a direct relationship exists between the cell’s metabolic phenotype and drug response. We first performed a metabolic profiling of the seven human melanoma cell lines using the Seahorse MitoStress test and compared them with that of primary human melanocytes. We used the Seahorse XF96 flux analyzer to measure in real-time the oxygen consumption rate (OCR) and extracellular acidification rate (ECAR) which are indirect measures of mitochondrial respiration (OXPHOS) and glycolytic activity, respectively [37]. The ratio of OCR to ECAR can indicate cellular preference for OXPHOS versus glycolysis when mitochondria are coupled for oxygen consumption and energy generation through complex V activity [38]. The measurements were performed under basal conditions and during the successive addition of metabolic stressors. Our results showed that the basal OCR and ECAR rates varied across cell lines, but the general response to the energy stressors remained consistent, including a decrease in OCR and increase in ECAR when treated with complex V inhibitor (oligomycin) and an increase in OCR and increase in ECAR when treated with the well-known uncoupling agent FCCP (Figure 2A). Specifically, the subsequent decreases in OCR after the addition of oligomycin suggest varying dependencies on ATP-linked respiration, and the variable FCCP-stimulated OCR increase indicates each cell line responded differently to increased energy demand. We observed that all seven melanomas have relatively lower basal OCR/ECAR ratios compared with primary human melanocytes (Figure 2B). However, all cells displayed basal OCR/ECAR greater than 2.0, indicating that these cells were actively utilizing mitochondrial respiration (OXPHOS). Interestingly, we found a significant inverse correlation between the OCR/ECAR ratios and the measured IC_50_ values for both SR4 (r = -0.808, *p* = 0.028) and niclosamide (r = -0.821, *p* = 0.024) (Figure 2C), suggesting that increased OXPHOS is indicative of greater sensitivity to uncoupling.

### SR4 and niclosamide uncouple mitochondria and induce metabolic reprogramming in melanoma

**Figure 3:**
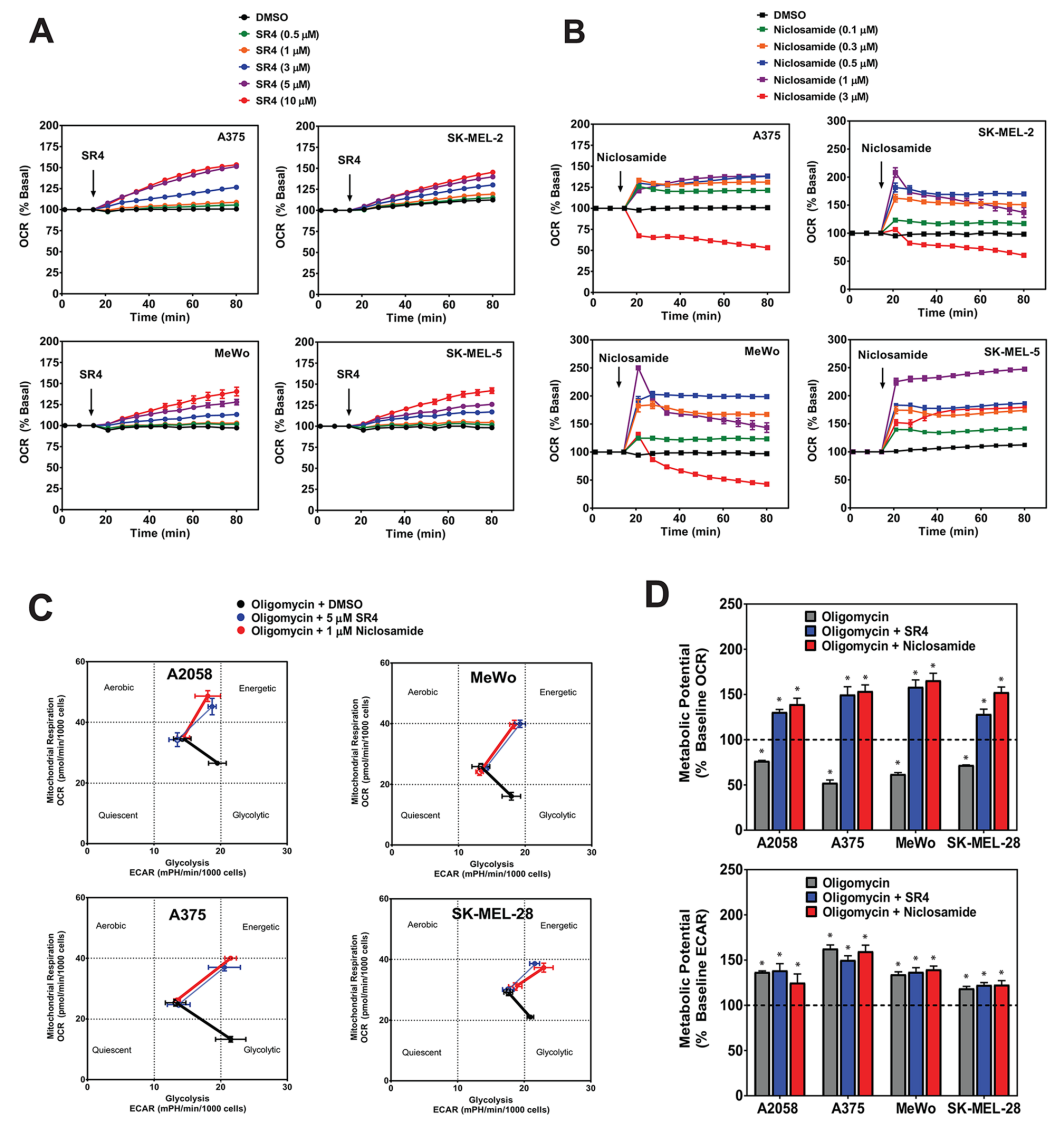
SR4 and niclosamide uncouple mitochondria and increase metabolic potential in melanoma cells **(A)** OCR of cultured melanoma cells treated with various concentrations of SR4 (0-10 μM) or **(B)** niclosamide (0-3 μM) as measured using Seahorse XF96 flux analyzer. **(C)** Metabolic phenogram analysis of OCR and ECAR by Seahorse Cell Energy Phenotype test. Cells were treated with 1 μM oligomycin with or without SR4 (5 μM) or niclosamide (1 μM) and the change in metabolic phenotype was assessed after 1 h treatment. **(D)** Metabolic OCR and ECAR potential of cells treated with oligomycin alone or oligomycin + SR4 or niclosamide as determined from the Cell Energy Phenotype test. Dotted line indicates baseline levels. Data in all three figures are means from 6-8 wells ± SEM, and are representative rates of two separate experiments. ^*^P < 0.05 vs. baseline levels.

Next, we examined the uncoupling effects of both SR4 and niclosamide in these melanoma cells. Increased OCR is a well-known consequence of mitochondria uncoupling [25]. Both compounds increased OCR in a time- and dose-dependent manner in all melanoma cells tested (Figure 3A and 3B, other cell lines not shown). Interestingly, compared with niclosamide which caused an abrupt increase in OCR at lower concentrations and a decrease in OCR at higher (>3 μM) concentrations, SR4 was able to maintain uncoupled respiration continuously at a high rate in all cell lines even at 10 μM. Thus, we used 5 μM SR4 and 1 μM niclosamide in the succeeding bioenergetic experiments to further investigate how the two compounds affect energy utilization and metabolic potential in these cancer cells. Metabolic potential is indicative of the cells’ ability, and preferred pathway, to respond to changes in energy demand due to stress. To this end, we used the XFp Cell Phenotype test (Seahorse) to assess the metabolic potential of cells treated with either SR4 or niclosamide. Acute exposure to either compound, even in the presence of ATP synthase inhibitor oligomycin (to inhibit stage 3 respiration), significantly shifted the baseline phenotype to the energetic phenotype characterized by increase in both OCR and ECAR metabolic potential in both BRAF^V600E^ mutant (A2058, A375, SK-MEL-28) and BRAF wild type (MeWo) cells (Figure 3C and 3D). Such an increase in OCR is primarily due to uncoupling, while the increase in ECAR is from the cells’ attempt to maintain their energy balance by using glycolysis to generate ATP [25]. This is in contrast with oligomycin where the cells have increased ECAR and a reduction in OCR, indicating a shift towards glycolysis. Taken together, these data suggest that uncoupling induces an acute energetic stress in melanoma cells to utilize both OXPHOS and glycolysis in response to increased energy demand.

### Uncoupling by SR4 and niclosamide creates energetic stress, activates AMPK and inhibits mTOR without any effects on ERK/MEK signaling pathways

**Figure 4:**
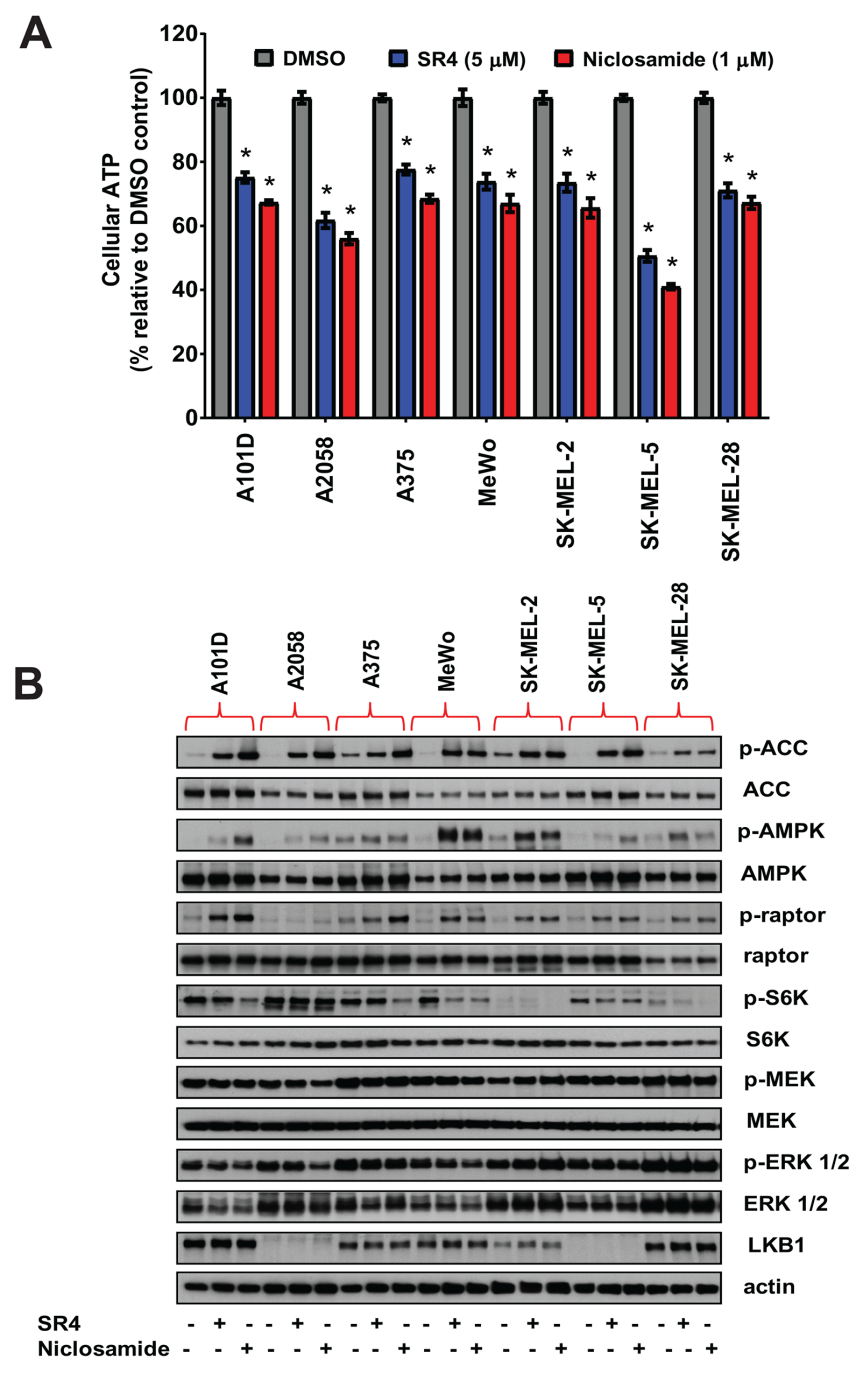
SR4 and niclosamide induce energetic stress and activate AMPK and downregulate mTOR in melanoma cells **(A)** Relative ATP levels in cultured melanoma cells after 1 h treatment with SR4 (5 μM) or niclosamide (1 μM). Total intracellular ATP was measured by bioluminescence assay and expressed as a percentage of vehicle (DMSO) control. ^*^P < 0.05 vs. DMSO control (n = 3). **(B)** Effects of SR4 and niclosamide on AMPK-mTOR and MAPK signaling. Representative Western blot analyses of melanoma cells treated with DMSO vehicle, SR4 (5 μM) or niclosamide (1 μM) for 4 h. For all blots, 5 μg of protein was loaded in each lane, resolved under electrophoresis and immunoblotted with antibodies against phosphorylated and total AMPK, ACC, raptor, S6K, ERK 1/2, MEK, LKB1 and β-actin, which served as an internal control. Data are representative results from two independent experiments.

To further investigate if metabolic phenotype changes are associated with cellular energy homeostasis, we measured the ATP levels in melanoma cells after treatment with SR4 or niclosamide for 1 h. As we have observed previously with SR4, the dissipation of the proton gradient and rapid collapse of the mitochondrial membrane potential induced by uncoupling leads to a rapid consumption of energy and oxygen without the generation of ATP [25]. As expected, both SR4 and niclosamide induced energetic stress as intracellular ATP levels were significantly reduced in all melanoma cell lines. Notably, we observed that A2058 and SK-MEL-5, the two most sensitive cell lines, had the lowest ATP levels after treatment with SR4 or niclosamide (Figure 4A). We further examined the metabolic effects of both SR4 and niclosamide in melanoma cells, specifically the AMPK-mTOR and MAPK signaling, since these pathways have been shown to be important mediators of melanoma growth and proliferation. The energy-sensor enzyme AMPK is activated in conditions of low energy, when the AMP:ATP ratio increases [12]. As expected and consistent with the reduction in intracellular ATP levels, both compounds activated AMPK as shown by the increase in AMPK phosphorylation in all melanoma cell lines (Figure 4B). AMPK phosphorylation was markedly higher in both BRAF wild type cells (Mewo and SK-MEL-2) compared with melanomas harboring the BRAF^V600E^ mutation. Among the BRAF^V600E^ mutants, A2058 and SK-MEL-5, two of the most sensitive cells to both uncouplers, and expressing either low or lacking the LKB1 protein, respectively, displayed the lowest AMPK phosphorylation (Figure 4B). Additionally, both SR4 and niclosamide increased the phosphorylation of acetyl-CoA carboxylase (ACC), one of the principal downstream targets of AMPK. However, the levels of ACC phosphorylation appeared to be independent of both BRAF and LKB1 status. Treatment of cells with either SR4 or niclosamide also inhibited mTOR signaling, as shown by increased phosphorylation of Raptor, a well-defined downstream target of AMPK, and decreased phosphorylation of the mTOR downstream effector p70S6 kinase (S6K). Both SR4 and niclosamide have very little or no effect on both ERK and MEK signaling as indicated by almost similar levels of phosphorylated proteins compared with vehicle control.

### SR4 and niclosamide inhibit tumor growth in A375 xenograft model

**Figure 5:**
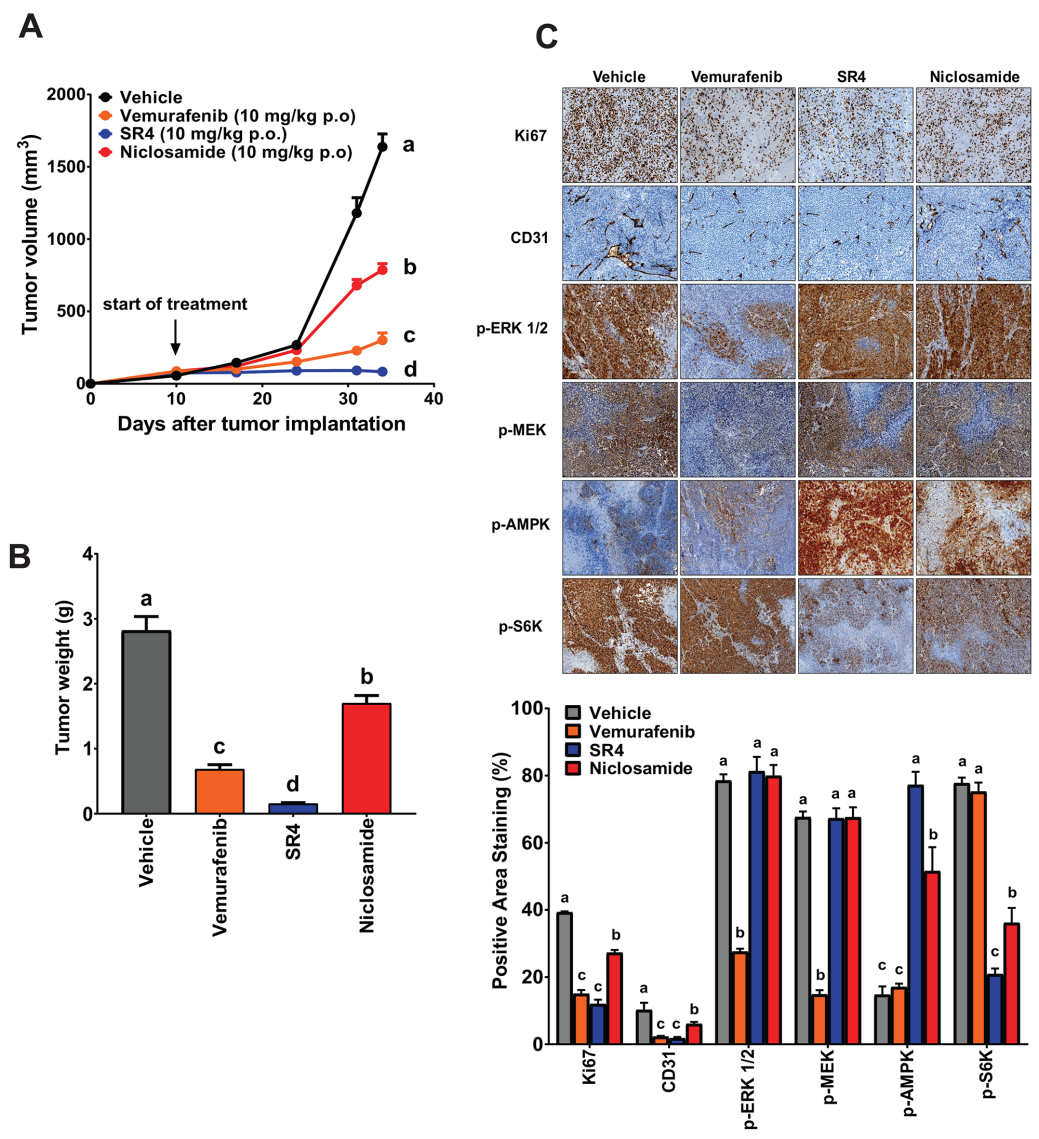
SR4 and niclosamide inhibit BRAF^V600E^ mutant melanoma *in vivo* **(A)** Tumor volume time course in nude mice with BRAF^V600E^ mutant melanoma treated with test compounds. Athymic *nu/nu* mice bearing A375 xenografts (1 × 10^6^ cells) were treated with vehicle, SR4 (10 mg/kg/day), niclosamide (10 mg/kg/day) or vemurafenib (10 mg/kg/day) p.o. when tumor volume reached 50 mm^3^. Tumor volume was measured at the indicated time points. Data are means ± SEM (n = 6). **(B)** Weight of dissected tumors from each treatment group. Data are means ± SEM (n = 6). **(C)** Representative immunohistochemical images of tumor tissue sections stained with specific antibodies against Ki-67, CD31, p-AMPK, p-ERK 1/2, p-MEK and p-S6K. Original magnification 100x. Bottom panel represents quantification of positive antibody staining (mean ± SEM, n = 6-9 fields per group). In all figures, means without common letters are significantly different (*P* < 0.05).

We used the human A375 BRAF^V600E^ mutant melanoma xenograft model to assess the *in vivo* efficacy of the two uncouplers in comparison with the BRAF inhibitor vemurafenib. As shown in Figure 5A and 5B, vemurafenib, SR4 and niclosamide, all given at 10 mg/kg daily via oral gavage, significantly reduced tumor growth and tumor mass in A375 xenograft mice. Specifically, SR4 was superior to vemurafenib and almost completely suppressed tumor growth (94.9% *vs*. 81.6%) and markedly reduced tumor mass (93.6% *vs*. 71.7%). Surprisingly, niclosamide was the least effective with 52.0% and 31.8% reductions in tumor volume and tumor weight, respectively. There was no indication of toxicity in all treatment groups as we did not observe significant effects on body weight and body temperature, and no major pathological changes in key organs such as the liver, heart and kidney were detected (Supplementary Figures 1A-1C). Immunohistochemical analysis of tumor sections revealed that vemurafenib, SR4 and niclosamide inhibited the proliferation marker Ki-67 and the angiogenesis marker CD31 (Figure 5C). As expected, vemurafenib significantly inhibited the MEK/ERK signaling as demonstrated by reduced antibody stainings of both p-ERK and p-MEK. However, it did not affect AMPK-mTOR signaling as the intensity of p-AMPK and p-S6K were similar with vehicle control. In contrast, both SR4 and niclosamide did not reduce both p-ERK and p-MEK levels, but increased p-AMPK and decreased p-S6K as observed similarly in the *in vitro* studies.

### SR4 and niclosamide overcome acquired resistance *in vitro*

**Figure 6:**
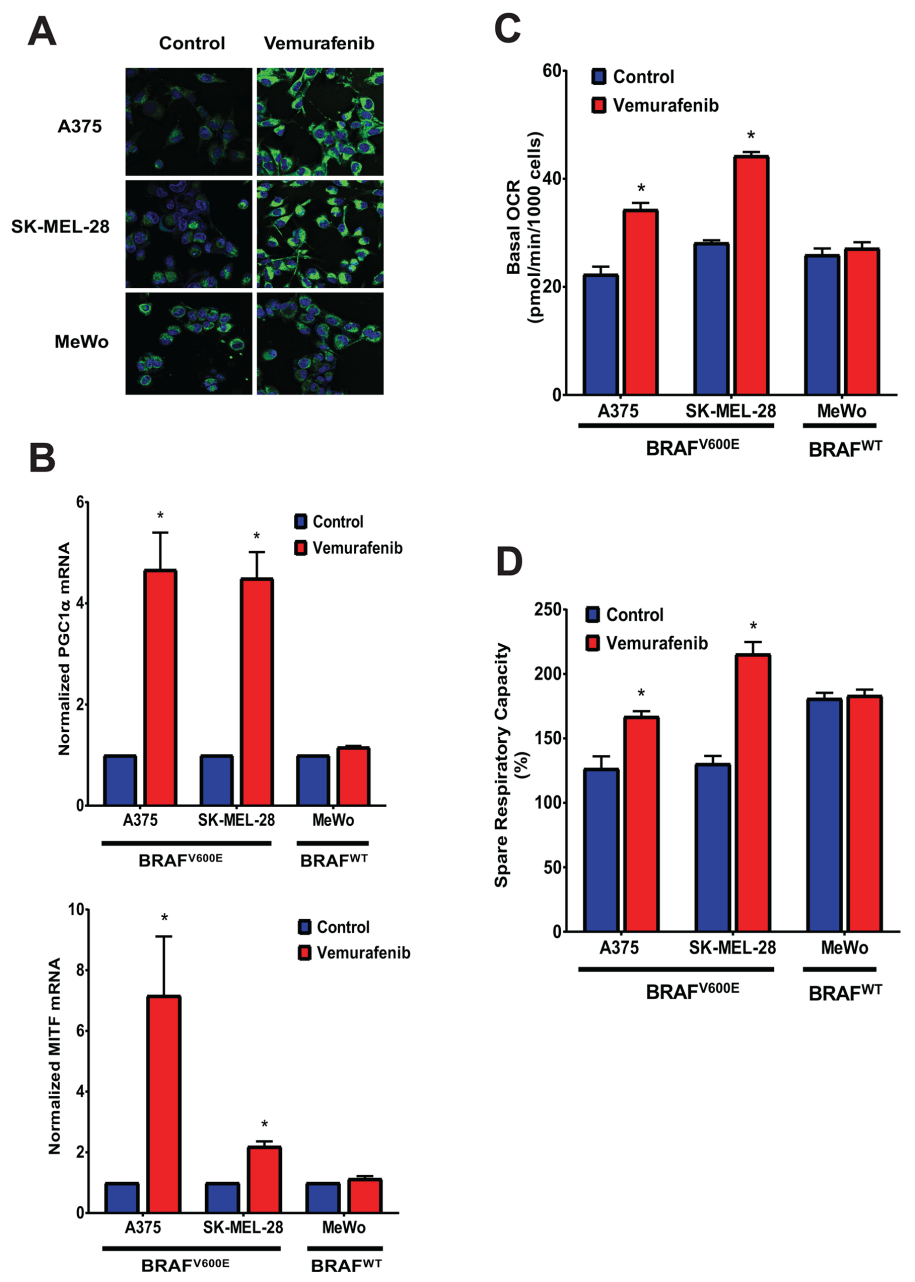
Vemurafenib increases mitochondrial density, MITF and PGC1α expression, and mitochondrial respiration in BRAF^V600E^ mutant melanomas **(A)** Representative Mitotracker Green fluorescence staining of BRAF^V600E^ mutant and BRAF wild type melanoma cells chronically treated with vemurafenib. **(B)** MITF and PGC1α mRNA expression of BRAF^V600E^ mutant and BRAF wild type melanoma cells chronically treated with vemurafenib. Values represent mean ± SEM of two independent experiments performed in triplicates. ^*^P < 0.05 vs. control. **(C)** Basal OCR and **(D)** respiratory capacity measurements from Seahorse XF96 flux analyzer of BRAF^V600E^ mutant and BRAF wild type melanoma cells chronically treated with vemurafenib. Data are means from 6-8 wells ± SEM, and are representative rates of two independent experiments. ^*^P < 0.05 vs. control.

**Figure 7:**
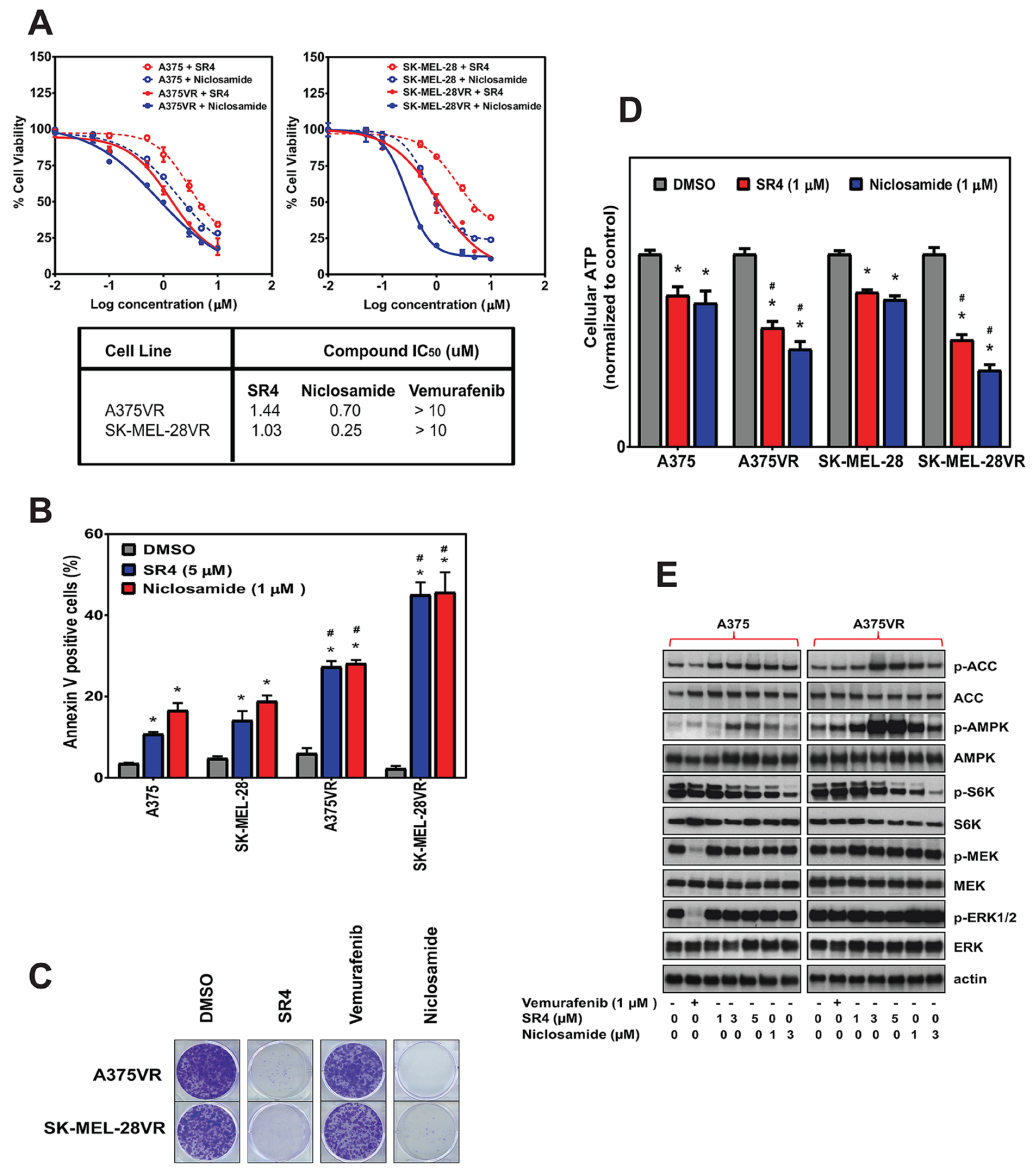
SR4 and niclosamide inhibit proliferation of vemurafenib-resistant melanoma *in vitro* **(A)** Comparative dose response curves and IC_50_ values of parental (A375, SK-MEL-28) and vemurafenib-resistant (A375VR, SK-MEL-28VR) melanoma cells treated with SR4, niclosamide and vemurafenib. Cell viability was measured by Cell Titer Glo assay after 48 h. IC_50_ values were calculated using GraphPad prism (*n* = 3). **(B)** Annexin V staining of parental and vemurafenib-resistant cells after treatment with SR4 (5 μM) and niclosamide (1 μM) for 48 h. Data are mean ± SEM (n = 3). ^*^P < 0.05 vs. DMSO control, ^#^*P* < 0.05 vs. parental cell. **(C)** Representative colony formation assays of vemurafenib-resistant cells following treatments with DMSO control or 1 μM each of SR4, niclosamide and vemurafenib. **(D)** Relative ATP levels in parental and vemurafenib-resistant cells after treatment with SR4 (5 μM) or niclosamide (1 μM) after 1 hr. Total intracellular ATP was measured by bioluminescence assay and expressed as a percentage of vehicle (DMSO) control. ^*^P < 0.05 vs. DMSO control, ^#^*P* < 0.05 vs. parental cell (n = 3). **(E)** Representative Western blot analyses of vemurafenib-resistant A375VR cells treated with DMSO vehicle, SR4 or niclosamide for 4 h. For all blots, 5 μg of protein was loaded in each lane, resolved under electrophoresis and immunoblotted with antibodies against phosphorylated and total AMPK, ACC, raptor, S6K, ERK 1/2, MEK, LKB1 and β-actin, which served as an internal control. Data are representative results from two separate experiments.

To investigate whether both uncouplers could overcome acquired resistance to BRAF inhibitor, two BRAF^V600E^ mutant (A375, SK-MEL-28) and BRAF wild type (MeWo) cells were chronically treated with increasing concentrations of vemurafenib (0.05, 0.1, 0.5, 1, 5 and 10 μM) through several passages. The two BRAF^V600E^ mutant cell lines (A375VR and SK-MEL-28VR) successfully acquired resistance to vemurafenib. Cells were considered resistant when they can be continuously cultured at 10 μM concentration of vemurafenib (it took approximately 1 and 2 months for SK-MEL-28VR and A375VR, respectively, to become resistant). Staining with the fluorescent dye MitoTracker Green showed that these vemurafenib-resistant cells became elongated and displayed an increase in mitochondrial content compared with parental cells (Figure 6A). These drug-resistant cells also exhibited increased mRNA expression of PGC1α and MITF (Figure 6B). In addition, metabolic characterization using the Seahorse XF96 flux analyzer showed that these vemurafenib-resistant cells have transformed metabolic profile with increased mitochondrial respiration as demonstrated by higher basal OCR values (Figure 6C) and significantly greater respiratory capacity (Figure 6D) compared with parental lines. The mitochondria density, PGC1α and MITF mRNA expressions, and basal OCR values of the BRAF wild type MeWo were not affected by chronic vemurafenib treatment. Exposure of these vemurafenib-resistant cells to either SR4 or niclosamide showed that these cells were more sensitive to both uncouplers compared with the parental cells as indicated by lower IC_50_ values (Figure 7A) and higher rates of apoptosis (Figure 7B). Moreover, both compounds completely inhibited colony formation in these drug-resistant cells (Figure 7C). As expected, vemurafenib treatment failed to induce cell death and showed no inhibitory effects on colony formation.

To determine the metabolic effects of SR4 and niclosamide in these vemurafenib-resistant cells, we again used the XFp Cell phenotype test and Seahorse flux analyzer. In A375VR cells, treatment with SR4 or niclosamide significantly shifted the cells’ metabolism to an energetic phenotype with increased OCR and ECAR (Supplementary Figure 2A), resulting to a significantly higher metabolic stress potential as seen earlier with the A375 parental line treated at the same duration (Supplementary Figure 2B). Furthermore, exposure to both compounds also produced significantly greater ATP loss in A375VR and SK-MEL-28VR cells than their counterpart parental lines (Figure 7D). To further investigate the effects of both uncouplers in vemurafenib-resistant cells, we analyzed the signaling pathways using Western blots. A375VR cells treated with vemurafenib showed reactivation of the MAPK signaling pathway as demonstrated by increased ERK and MEK phosphorylation. Both SR4 and niclosamide showed no effects on ERK and MEK signaling as phosphorylation levels of both proteins remained the same in both A375 parental and A375VR cells. However, both compounds increased phosphorylation of AMPK and ACC noticeably higher than in A375VR cells, while also decreasing S6K phosphorylation (Figure 7E).

### SR4 and niclosamide inhibit vemurafenib-resistant tumor *in vivo*

**Figure 8:**
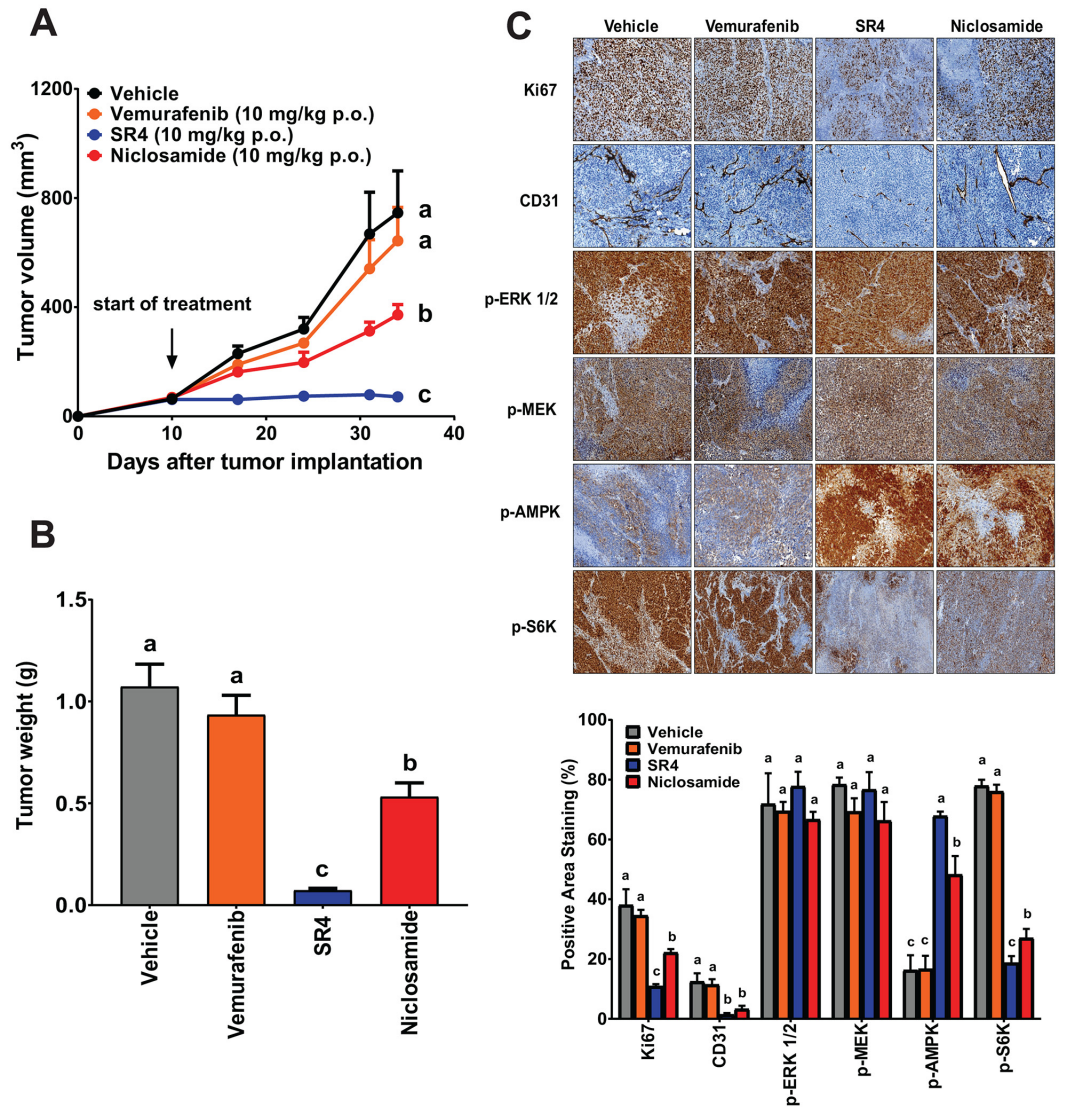
SR4 and niclosamide inhibit vemurafenib-resistant melanoma *in vivo* **(A)** Tumor volume time course in nude mice bearing vemurafenib-resistant melanoma treated with test compounds. Athymic *nu/nu* mice bearing A375VR xenografts (1 × 10^6^ cells) were treated with vehicle, SR4 (10 mg/kg/day), niclosamide (10 mg/kg/day) or vemurafenib (10 mg/kg/day) p.o. when tumor volume reached 50 mm^3^. Tumor volume was measured at the indicated time points. Data are means ± SEM (n = 6). **(B)** Weight of dissected tumors from each treatment group. Data are means ± SEM (n = 6). **(C)** Representative immunohistochemical images of tumor tissue sections stained with specific antibodies against Ki-67, CD31, p-AMPK, p-ERK 1/2, p-MEK and p-S6K. Original magnification 100x. Bottom panel represents quantification of positive antibody staining (mean ± SEM, n = 6-9 fields per group). In all figures, means without common letters are significantly different (*P* < 0.05).

We used the A375VR cells to evaluate the efficacy of SR4 and niclosamide against vemurafenib-resistant melanoma. These vemurafenib-resistant cells grew relatively slower than the parental A375 cells in nude mice, consistent with their growth rates *in vitro* (Supplementary Figure 3). Nonetheless, SR4 again demonstrated better potency than niclosamide in reducing tumor growth (94.0 *vs*. 50.0 %) and tumor mass (93.3% *vs*. 50.4%) (Figure 8A and 8B). As expected, vemurafenib treatment showed no antitumor activity as it displayed similar tumor growth rate and tumor weight compared with the vehicle control. Immunohistochemical analyses also showed that vemurafenib had no effect on Ki-67, CD31, p-ERK and p-MEK expressions in tumor sections from these animals. Consistent with the *in vitro* studies and mirrored in the results of A375 xenograft study, SR4 and niclosamide increased the phosphorylation levels of AMPK and decrease S6K, but did not affect ERK and MEK phosphorylation (Figure 8C). In addition, both uncouplers inhibited Ki-67 and CD31 as shown by reduced antibody staining in tumor sections of A375VR xenograft mice.

## DISCUSSION

Given the limited treatment options available for advanced stages of melanoma, the development of novel targeted therapy has immense translational significance in contemporary melanoma research. Mitochondrial targeted drugs represent a potential new anti-cancer therapy due to the significant differences in structure, function, and bioenergetics of mitochondria between cancer and normal cells [39]. Compounds that are lipophilic enough to reach the mitochondrial membrane could potentially induce cell death by means of mitochondrial mechanisms. In this study, we demonstrated that both small molecule SR4 and niclosamide create energetic stress in melanoma irrespective of BRAF/NRAS status by uncoupling mitochondrial OXPHOS, decreasing intracellular ATP production, activating the energy sensor and metabolic tumor suppressor AMPK, and downregulating the mTOR pathway, leading to inhibition of tumor proliferation *in vitro* and in xenograft mice models. This concept of inhibiting OXPHOS and/or AMPK activation to inhibit melanoma proliferation *in vitro* and *in vivo* has been demonstrated recently using pharmacological direct AMPK activators such as 5-aminoimidazole-4-carboxamide ribonucleoside (AICAR) [40, 41] and GSK621 [42], and OXPHOS inhibitors such as biguanides (metformin and phenformin) [40, 41, 43, 44] and BAY- 87-2243 [45]. Thus, our current data suggest that utilizing mitochondrial uncouplers to inhibit OXPHOS and indirectly activate AMPK could be an alternative first-line of treatment for melanoma irrespective of oncogenic driver mutations. This treatment strategy offers a distinct advantage over MAPK inhibitors that can only be used for melanomas harboring the BRAF mutations, consequently limiting the population of patients that is eligible for their use.

Metabolic reprogramming and altered bioenergetics have been recognized in recent years as a hallmark of cancer. In contrast to normal differentiated cells which rely primarily on OXPHOS to generate the energy needed for cellular processes, it has been widely accepted that most cancer cells rely on aerobic glycolysis (Warburg effect) [46]. The Warburg effect hypothesized that existing mitochondrial dysfunction disrupts the OXPHOS pathway, and therefore, cancer cells have to switch from OXPHOS to glycolysis for ATP generation. However, the Warburg effect has been challenged lately due to findings that upregulated glycolysis in many cancers is not accompanied by detectable mitochondrial defects or OXPHOS disruptions, and that there is no OXPHOS-to-glycolysis switch [47]. In fact, recent investigations show that the shift from glycolysis to oxidative metabolism is required for certain steps of tumor progression [48], suggesting that mitochondrial function are crucial for metabolic adaptations. A growing body of evidence shows that OXPHOS is preserved in many cancer cells including melanoma even under hypoxic conditions [22], and these cells are able to generate ATP through OXPHOS fueled by fatty acids and amino acids such as glutamine [49–51]. Indeed, some patient-derived metastatic melanomas display the OXPHOS metabolism and have high OCR values [23, 24]. Recent studies estimated that between 35% and 50% of BRAF-mutant and wild-type cell lines and patient samples can be characterized as “high-OXPHOS” phenotype predominantly driven by PGC1α [18, 52, 53]. These high OXPHOS melanomas exhibit aggressive clinical behavior and confer *de novo* resistance to both MAPK inhibition and oxidative stress [53]. In the current study, we screened seven human melanoma cells lines representing distinct oncogenic drivers (two BRAF wild type, five BRAF^600E^ and one NRAS mutant) for their basal metabolic phenotype and observed that all of them displayed OCR/ECAR ratio indicative of OXPHOS activity. More importantly, we found a strong inverse correlation between the OCR/ECAR ratios and the sensitivity of the cells to both uncouplers, *i.e*., those cells with higher OCR/ECAR (=higher OXPHOS) displayed lower IC_50_ for both compounds. We speculate that these observed differences in OCR/ECAR values might be correlated with the levels of PGC1α of each cell line. Previous studies reported that A375, SK-MEL-2 and SK-MEL-28 have low levels of PGC1α, whereas SK-MEL-5 and MeWo were classified as high PGC1α melanomas based on their mRNA and protein expression levels of this transcriptional coactivator [53]. Interestingly, we observed that A375, SK-MEL-2 and SK-MEL-28 displayed three of the lowest OCR/ECAR values and were the least sensitive to both uncouplers, while high PGC1α cells such as SK-MEL-5 and MeWo displayed greater sensitivity to both uncouplers and have relatively higher OCR/ECAR than the other cell lines. Consistent with our current findings, the greater vulnerability of cancer cells with higher OCR/ECAR ratio to energy stressors like mitochondrial inhibitors and biguanide drugs has been similarly observed more recently [54], and suggests the possibility of using OXPHOS genes/proteins as biological markers in the diagnosis, stratification and treatment of melanoma patients with these OXPHOS modulators.

An important metabolic property of tumor cells is the ability to suppress the energetic stress checkpoint modulated by the LKB1-AMPK signaling pathway. Several studies suggest a molecular link between MAPK signaling and the LKB1-AMPK pathway in melanoma. BRAF^V600E^ oncogenic mutation has been shown to impair AMPK activation in melanoma by promoting inhibitory phosphorylation on LKB1 by ERK 1/2 and that this AMPK inhibition is critical for melanoma cell proliferation [14, 16]. In this study, we observed that AMPK activation by both uncouplers was significantly less in BRAF^V600E^ melanoma compared with BRAF wild type as seen in the Western blot analyses. Among the five BRAF^V600E^ mutants screened in our studies, A2058 and SK-MEL-5 were the most sensitive to both uncouplers, despite the fact their AMPK phosphorylation levels were the lowest. As noted previously, these two melanoma cells are LKB1 mutant and LKB1 null, respectively. Cancer cells with a defective LKB1/AMPK pathway are less able to restore ATP levels in response to energetic stress, and thus, are more susceptible to cell death than cells with a functional LKB1/AMPK [13]. Thus, it was not surprising that these two cell lines also had the greater loss in intracellular ATP after treatment with the uncouplers. Although LKB1 mutant melanomas appear to be excellent therapeutic targets for uncouplers and other energy stressors, less than 10% of melanomas have this mutation [55].

Additionally, we observed that both SR4 and niclosamide have no effect on ERK/MEK signaling pathway in all melanoma cell lines we tested despite increased AMPK activation. Previous research showed that wild type BRAF is phosphorylated at Ser^729^ by AMPK, and this phosphorylation promotes the association of BRAF with 14-3-3 proteins and disrupts its interaction with the KSR1 scaffolding protein, leading to attenuation of the MEK-ERK signaling [56]. Furthermore, it is thought that oncogenic BRAF is resistant to AMPK-mediated inhibition, so ERK signaling cannot be attenuated by AMPK in BRAF mutant melanoma [56]. Studies using the biguanides metformin and phenformin, both indirect AMPK activators, showed minimal effects on ERK dephosphorylation in both BRAF wild type and BRAF^V600E^ mutant, as well as NRAS mutant melanomas [44, 57]. In contrast, several studies using other indirect AMPK agonists demonstrated that activation of AMPK was associated with reduced ERK signaling even in BRAF^V600E^ mutant cells [45, 58]. These mixed results indicate that AMPK-associated ERK deactivation in melanoma could be cell and stressor specific, or there are still several unidentified negative feedback loops that are operating between BRAF, MAPK and LKB1/AMPK pathways as suggested recently [12]. Nonetheless, the strategy of simultaneously targeting both the MAPK pathway and OXPHOS using a combination of MAPK inhibitors and OXPHOS inhibitors/AMPK agonists has been highly effective in several preclinical studies [44, 45, 57], and are currently being investigated in phase I/II metastatic melanoma clinical trials (NCT01638676, NCT02143050 NCT03026517). Whether the combination of MAPK inhibitors and mitochondria uncouplers could result to similar synergistic/additive effects in melanoma remains to be investigated.

It was proposed more recently that acquired resistance to BRAF inhibitors results from two successive steps, an early metabolic reprogramming followed by subsequent mutation(s) promoting cell proliferation [59]. Inhibition of BRAF mutations using MAPK inhibitors suppresses glycolysis [60] leading to temporarily suppression of melanoma growth, but also promotes metabolic switch to OXPHOS phenotype via induction of MITF and PGC1α expression [17, 18, 19, 20, 51]. Indeed, we observed increased mRNA expression of both MITF and PGC1α in the two BRAF^V600E^ mutant cell lines chronically treated with vemurafenib, concomitant with increased mitochondria density. In addition to the reactivation of the MEK/ERK, these drug resistant cell lines also exhibited increased OXPHOS as indicated by significantly higher basal OCR values and greater respiratory capacity compared with parental lines. Consequently, these drug-resistant cells were more sensitive to the uncoupling effects of SR4 or niclosamide. We also found that both compounds produced significantly larger ATP loss and greater activation of AMPK in these cells. In connection with these findings, we also observed similar upregulation of OXPHOS and increased sensitivity to SR4 or niclosamide of melanoma cells chronically treated with the MEK inhibitor trametinib or with the vemurafenib/trametinib combination (data not shown). Collectively, our data support other recent studies showing that melanomas with acquired resistance to MAPK inhibitors are more vulnerable to the effects of energy stressors [17, 45, 51]. Thus, aside from being an alternative first-line treatment for melanoma independent of oncogenic driver mutations, mitochondria uncouplers such as SR4 and niclosamide may also find utility for those patients failing MAPK inhibitor therapy. Given the diverse resistance mechanisms to both MEK/ERK inhibition, a drug that can inhibit MAPK inhibitor-resistant melanomas, regardless of the resistance mechanism(s), offers a therapeutic advantage in the clinic.

Our *in vitro* data clearly shows that niclosamide has more potent anti-cancer activities than SR4 across all seven treatment-naïve and two vemurafenib-resistant melanoma cell lines we investigated. These findings also mirrored the NCI-DTP60 screening results where the growth inhibition (GI_50_) of niclosamide was lower than SR4 in all melanoma cells and other human cancers, with the exception of two colorectal cancer cell lines (Supplementary Table 1). However, we found that SR4 was superior to niclosamide when given a similar oral daily dose of 10 mg/kg b.w. in preventing tumor growth in treatment naïve BRAF^V600E^ and vemurafenib-resistant mice xenograft studies. Even though both compounds share similar chemical properties (low molecular weight, poor water solubility), the higher efficacy of SR4 could be attributed to its better pharmacokinetic (PK) profile. Previous studies in rats [61] showed that when administered at 5 mg/kg orally, niclosamide exhibited a short half-life, (T_1/2_ = 6.0 ± 0.8 h) and was rapidly absorbed within 30 min (T_max_ = 0.4 ± 0.1 h) with a maximum concentration (C_max_) of 354 ± 152 ng/ml. The area under curve (AUC) and bioavailability were 429 ± 100 ng/ml × h and 10%, respectively. In mice orally dosed with niclosamide at 200 mg/kg [30], almost similar PK parameters were observed (T_1/2_ = 3.2 h; T_max_ = 15 min; C_max_ = 893 ng/ml; AUC = 1011 ng/ml × h). In contrast, SR4 dosed at 10 mg/kg orally in rats exhibited similar half-life (6.2 ± 1.9 h) and C_max_ (454 ± 235 ng/ml), but higher T_max_ (5.3 ± 2.3 h) and AUC (4263 ± 2563 ng/ml × h) (unpublished data). Thus, the low solubility, low bioavailability and rapid clearance of niclosamide could result to low overall plasma drug exposure compared with SR4 when dosed orally. Indeed, relatively higher doses (∼ 100 to 200 mg/kg body weight) of niclosamide were needed in order to demonstrate significant inhibition of tumor growth in xenograft mice [30, 31].

Like most drug targets, uncoupling OXPHOS and activating AMPK might cause some undesirable side effects. Using pharmacological agents to uncouple mitochondria could be a risky treatment, because it might compromise energy homeostasis in other tissues, such as the heart and brain, causing irreversible damage. For uncouplers to work safely, the compound should be selectively targeted to cancer cells and cause uncoupling that increases very little when its concentration rises, potentially widening the therapeutic window. Interestingly, the lipophilic property of SR4 (logP = 5.53) and niclosamide (logP = 5.40) allows either of them to be selectively targeted to and accumulate in cancer cells which have more hyperpolarized mitochondrial membrane potential than normal cells [62]. In our *in vitro* assays, both SR4 and niclosamide showed minimal toxicity to normal melanocytes, while animal xenograft studies showed no toxic effects on various organs such as the heart, kidney and liver. More importantly, both SR4 and niclosamide did not induce a hyperthermic effect, a well-known lethal side effect of some uncouplers. In previous animal studies, oral administration of SR4 in C57B mice showed no overt toxic effects in most of the blood and metabolic parameters [27]. Similarly, data from toxicological studies showed that the LD_50_ of niclosamide for rats was greater than 5g/kg/day and administration of daily dose of 2 g/kg/day for four weeks produced no toxicity [61]. Some of the reported detrimental effects of AMPK activation include increased food intake and body weight gain via activation of the hypothalamic AMPK. Both SR4 and niclosamide had no effect on body weight in the current study, consistent with the earlier observations in other xenograft experiments [26, 27, 31]. These suggest that the risk associated with the SR4 or niclosamide-induced mitochondria uncoupling is probably low.

**Figure 9:**
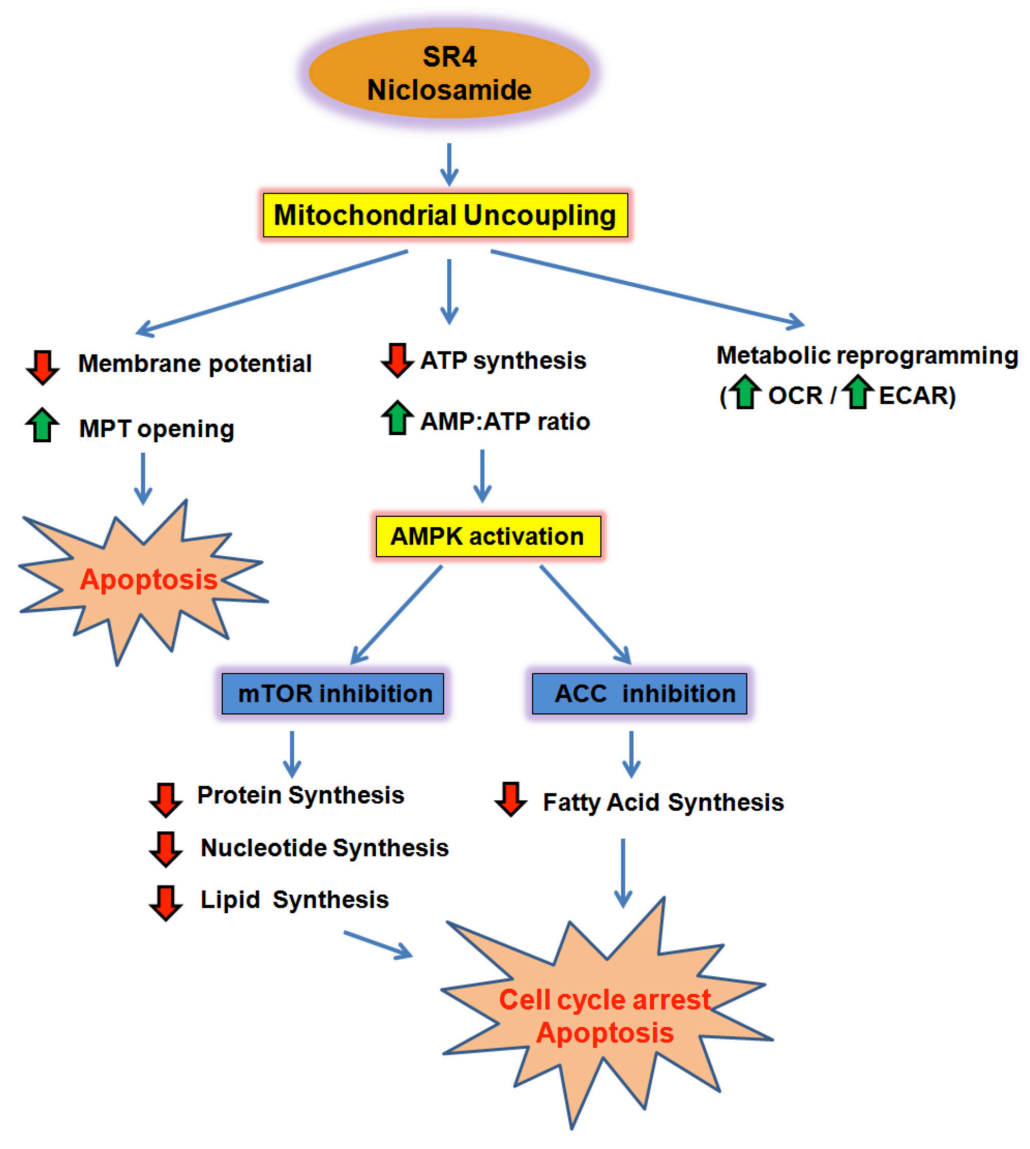
Summary model of SR4 and niclosamide effects in melanoma SR4 and niclosamide uncouple mitochondrial OXPHOS, inhibit ATP synthesis and create energetic stress in melanoma. Reduction in cellular ATP and increase in AMP:ATP ratio indirectly activate the energy sensor enzyme AMPK. AMPK activation prevents melanoma growth and proliferation by inhibiting mTOR activity and lipid synthesis, promoting cell cycle arrest and apoptosis. Uncoupling also results in the collapse of mitochondria membrane potential, leading to opening of the mitochondria transition pore (MPT) and mitochondrial-dependent apoptosis. In addition, uncoupling induces metabolic reprogramming of melanoma cells, as it promotes an energetic phenotype characterized by increased oxygen consumption and glycolysis. Red arrows represent decreased intensity, while green arrows indicate an increase.

In summary, we demonstrated that both small molecule SR4 and niclosamide have anti-proliferative and pro-apoptotic activities against melanoma irrespective of BRAF and NRAS status. Both compounds uncouple OXPHOS and induce energetic stress, leading to modulation of the AMPK-mTOR signaling pathway (Figure 9). We found that melanomas with higher OCR/ECAR, with LKB1 mutation, or with acquired resistance from MAPK inhibitors displayed greater sensitivity to both uncouplers. Importantly, SR4, and to a lesser extent niclosamide, significantly inhibited tumor growth of both treatment naïve and vemurafenib-resistant melanoma in mice without toxic effects. These findings suggest that uncouplers such as SR4 and niclosamide have the potential to be developed as a first line treatment against metastatic melanoma as well as adjuvant therapy for patients failing MAPK inhibitors.

## MATERIALS AND METHODS

### Chemicals and reagents

SR4 was synthesized according to a previously validated protocol at the Chemical GMP Synthesis Facility, Translational Medicinal Chemistry Laboratory, Beckman Research Institute of the City of Hope [25]. The BRAF inhibitor vemurafenib was purchased from ApexBio (Houston, TX, USA). Antibodies against AMPKα, phospho-AMPKα (Thr^172^), ACC, phospho-ACC (Ser^79^), p70S6 kinase (S6K), phospho-S6K (Thr^389^), ERK 1/2 (p44/42 MAPK), phospho-ERK (Thr^202^/Tyr^204^), Raptor, phospho-Raptor (Ser^792^), MEK, phospho-MEK (Ser^217^/Ser^221^), p38, phospho-p38 (Thr^180^/Tyr^182^), LKB1 and β-actin were obtained from Cell Signaling Technology (Danvers, MA, USA). Unless otherwise noted, all other chemicals and reagents, including niclosamide, were purchased from Sigma-Aldrich (St. Louis, MO, USA).

### Cell culture

The human melanoma cell lines A101D, A375, A2058, MeWo, SK-MEL-2, SK-MEL-5, SK-MEL-28 were obtained from ATCC and were cultured in RPMI-1640 containing 10% fetal bovine serum and 1% penicillin/streptomycin antibiotics (ATCC, Manassas, VA, USA) at 37°C in a humidified atmosphere of 5% CO_2_. Cells were sub-cultured every 2–3 days at ∼70–80% confluence. Primary adult human melanocytes were purchased from Sciencell (Carlsbad, CA, USA) and were cultured in melanocyte growth media. All melanoma cells were authenticated by STR analysis and were free of *Mycoplasma* infection as tested by the universal *Mycoplasma* detection kit (ATCC).

### Induction of *in vitro* acquired vemurafenib resistance

To generate vemurafenib resistant-melanoma *in vitro*, two BRAF^V600E^ cell lines (A375 and SK-MEL-28) were chronically exposed to increasing concentrations of vemurafenib until they grew steadily above the IC_50_ (∼ 1 and 2 months for SK-MEL-28 and A375, respectively) and were then maintained in medium containing 10 μM vemurafenib prior to drug testing.

### Cell viability and apoptosis assays

Cells were seeded into 96-well white polystyrene microplates at density of 5,000 cells/well and were incubated overnight at 37°C prior to drug treatment. Drugs were diluted in cell culture medium and added to each well at the indicated concentrations (triplicate wells/drug concentration). Cell viability was measured 48 h later using the Cell Titer-Glo^®^ Luminescent Cell Viability Assay. Cell viability of drug-treated cells was expressed as a percentage of control cells (i.e. cells treated with equivalent concentrations of the DMSO vehicle). The final concentration of DMSO exposed to the cells was <0.1% (v/v) for the duration of the experiment. The half maximal inhibitory concentration (IC_50_) for each test compound was calculated using the GraphPad Prism software. Apoptosis was determined by flow cytometry using the Annexin V-FITC apoptosis detection kit (Thermo Fisher Scientific, Waltham, MA, USA) as described by the manufacturer. Briefly, cells were plated in 6-well tissue culture plates (4 × 10^5^ cells/well) and incubated overnight. Medium was replaced with fresh RPMI containing the testing agents at the indicated final concentrations. After 48 h of treatment, cells were trypsinized and centrifuged at 1,000 rpm for 5 min. The cell pellets were then washed twice with 1 ml of ice-cold PBS, resuspended in 100 μl of binding buffer and were stained with 5 μl of Annexin V-FITC solution and 5 μl of PI solution for 20 min at room temperature in the dark. Then the samples were diluted with 400 μl of 1× binding buffer, processed for data acquisition, and analyzed by CyAn ADP flow cytometer. Approximately 30,000 cells were counted and analyzed for each sample. The percentage distribution of early and late apoptotic cells was calculated using Summit software (version 4.2, Cytomation Inc., Fort Collins, CO, USA). The experiments were repeated at least twice.

### Colony-formation assay

Colony-forming cell growth was obtained by growing melanoma cells in 12-well plates with indicated treatments for 10 days. The medium containing the DMSO vehicle or drug was refreshed every 3 days. Cell colonies were stained with 0.1% (w/v) crystal violet in PBS, dried, and photographed. The experiments were repeated at least 2-3 times.

### Cellular ATP measurement

Intracellular ATP levels of melanoma cells treated with the test compounds at the indicated time point were measured using the luminescent ATP detection assay kit (Abcam, Cambridge, MA, USA) and expressed as the percentage of vehicle (DMSO)-treated cells treated at the same time.

### Measurement of mitochondria density

For visualization of mitochondria, parental and vemurafenib-resistant cells were plated in a 12-well coverslip 10 mm glass diameter plate overnight at 37°C, 5 % CO_2_ incubator. Cells were then washed with PBS, before addition of live cell imaging solution (Molecular Probes^®^, Thermofisher) containing the Mitotracker Green FM (Invitrogen) and DNA dye Hoechst 33342 (Thermofischer) for 30 min at 37°C. Afterwards, the staining solution was removed and cells were washed twice with LCIS, and then 500 μl LCIS containing 10 mM glucose was added in each well. The live cells were then observed and photographed using a Zeiss LSM 880 laser scanning confocal microscope (Carl Zeiss Microscopy, Thornwood, NY, USA). Imaging data was subsequently analyzed and quantified using Image-Pro Premier (Version 9.2, Media Cybernetics, Rockville, MD, USA).

### Metabolic analysis using the Seahorse XF96 extracellular flux analyzer

Metabolic characterization of the melanoma cell lines and primary human melanocytes was first performed using the Seahorse XF96 flux analyzer (Seahorse Biosciences, North Billerica, MA, USA). In brief, cells were seeded in DMEM in 96-well tissue culture plates at a density of 5,000-10,000 cells per well and allowed to adhere for 24 h. Prior to the assay, the medium was changed to DMEM containing 10 mM glucose, 1 mM pyruvate, and 1 mM glutamine with 0.2% fatty acid/endotoxin-free BSA (w/v) (pH 7.4), and the cells were equilibrated for 30 min at 37°C. Real-time measurements of oxygen consumption rate (OCR) and extracellular acidification rate (ECAR) were measured under basal conditions and during successive addition of four metabolic modulators (MitoStress test): oligomycin (ATP synthase inhibitor), FCCP (the mitochondrial membrane permeabilizer/uncoupler) and rotenone/antimycin A (complex I and complex III inhibitor, respectively). Next, we examined the effects of both SR4 and niclosamide on mitochondria bioenergetics on the different melanoma cell lines. Various concentrations of the test compounds were added into the cells, and OCR and ECAR were measured using 3 minute measurement periods for 1 h. In another test, basal mitochondrial function and metabolic potential of melanoma cells upon exposure to test compounds were measured using the XFp Cell Energy Phenotype Test (Seahorse Bioscience). This assay simultaneously measures the two major energy producing pathways in live cells, mitochondrial respiration and glycolysis, with a single injection of pathway modulators, allowing rapid determination of energy phenotypes of cells and investigation of metabolic switching (metabolic potential). Briefly, cells were seeded at equal densities (5,000 cells/well) into XF96 tissue culture plates. Cell media was changed 24 h after cell seeding as described above. OCR and ECAR were measured under basal conditions and/or after injection of oligomycin with or without SR4 or niclosamide. Both MitoStress and Cell Energy Phenotype tests were also used to measure OCR and ECAR levels in vemurafenib-resistant (VR) melanoma cells exposed to DMSO vehicle, SR4 or niclosamide. In all these bioenergetic tests, all treatment conditions were analyzed with 6-8 wells/treatment and repeated at least twice. OCR/ECAR values were normalized to cell numbers.

### Protein extraction and Western blotting

Melanoma cell lines were grown to 80 % confluency and incubated with test compounds for 4 h, whereas control samples were treated with an equal volume of DMSO. Cellular proteins were extracted with cell lysis buffer (Cell Signaling Technology) and protein concentration was determined using the DC Protein Assay kit (Bio-Rad, Hercules, CA, USA). Equal amounts of proteins (5 μg) were loaded onto 4-15% Criterion TGX gels (Bio-Rad), resolved by SDS electrophoresis, and then transferred to nitrocellulose membranes for immunoblotting. Membranes were blocked with 5% skimmed milk in Tris-buffered saline containing 0.05% Tween 20 before incubation overnight at 4°C with primary antibodies against total and phosphorylated AMPK, ACC, ERK 1/2, MEK, raptor, p70S6K (S6K) and LKB1. Immunoreactive proteins were visualized by peroxidase-labeled secondary antibodies and ECL system (Western Lightning Chemiluminescence Reagent, Perkin-Elmer, MA, USA). Equal loading of proteins was confirmed by stripping and restaining the membranes with β-actin antibodies.

### Quantitative RT-PCR

Total RNA was isolated from cells using the RNEasy kit (Qiagen, Valencia, CA, USA). First strand cDNA was prepared using the High Capacity cDNA reverse transcription kit (Life Technologies). Pre-validated primer pairs for PGC1α, MITF and β-actin were purchased from Bio-Rad (PrimePCR™ PCR Primers). Quantitative RT-PCR was performed on three independent samples per cell line (or per treatment) using the ABI-7500 fast real-time PCR system and Power SYBR Green master mix ((Life Technologies). After initial incubation for 2 min at 50°C, the cDNA was denatured at 95°C for 10 min, followed by 40 cycles of PCR (95°C for 15 s, 60°C for 60 s). The relative mRNA levels of each gene were quantified using the comparative Ct method [63] with β-actin as an internal control.

### *In vivo* xenograft model studies

All animal experiments were carried out in accordance with a protocol approved by the Institutional Animal Care and Use Committee (IACUC) of the City of Hope National Medical Center. Athymic nude mice were obtained from Harlan Laboratories (Indianapolis, IN, USA). The melanoma xenograft mouse model was established by subcutaneous injection into the right flank of each animal with 1 × 10^6^ vemurafenib-sensitive A375 or vemurafenib-resistant A375 (A375VR) cell suspensions in 100 μl of PBS. Once the tumor volume reached around 50 mm^3^, the mice were randomized into vehicle control (4% DMSO in corn oil) and treatment groups (n = 6 per group), and the treatments were started. SR4 (10 mg/kg), niclosamide (10 mg/kg) and vemurafenib (10 mg/kg) was administered daily by oral gavage in 4% DMSO/corn oil vehicle. Body weight and tumor volume of the mice were recorded every 7 days using the formula *a* × *b*^2^ × 0.5, where *a* and *b* represent the larger and smaller tumor diameters [64].

### Histopathological examination of tumors for angiogenic, proliferative and differentiation markers

Formalin-fixed and paraffin-embedded tumor sections (4 μm thick) from vehicle control and drug-treated A375 and A375VR melanoma bearing mice were used for histopathologic analyses. The following antibodies were used for immunohistochemistry analyses: Ki-67, CD31, and phosphorylated ERK, MEK, S6K and AMPK. Photomicrographs at 100× magnification of stained sections were taken under bright field microscope (AX70, Olympus, Tokyo, Japan). Image quantification was done on at least 6-9 random fields representing three animal tumor sections in each treatment group using Image-Pro Premiere software.

### Statistical analyses

Statistical analyses were performed using Prism (GraphPad, San Diego, CA, USA). For comparison of more than two groups, one-way ANOVA followed by Tukey’s test was used. A two-tailed Student *t-test* was performed to compare between two groups. Correlation between OCR/ECAR ratio and IC_50_ values was calculated using Pearson correlation. All data are stated as mean ± SEM except noted. Differences between means are considered statistically significant when *P* < 0.05.

## SUPPLEMENTARY MATERIALS FIGURES AND TABLE





## Abbreviations

AUC: area under the curve
AICAR: 5-Aminoimidazole-4-carboxamide ribonucleotide
AMPK: 5’ AMP-activated protein kinase
BRAF: v-Raf murine sarcoma viral oncogene homolog B
DMSO: dimethyl sulfoxide
ECAR: extracellular acidification rate
ERK: extracellular signal–regulated kinase
FCCP: carbonyl cyanide-4-(trifluoromethoxy)phenylhydrazone
LKB1: liver kinase B1
MAPK: mitogen-activated protein kinase
MEK: mitogen-activated protein kinase kinase
MITF: microphthalmia-associated transcription factor
NRAS: neuroblastoma RAS viral oncogene homolog
OCR: oxygen consumption rate
OXPHOS: oxidative phosphorylation
PGC1α: peroxisome proliferator-activated receptor gamma coactivator 1-alpha
SR4: 1,3-bis (3,5-dichlorophenyl) urea
