# Supplementary material for: Bioenergetic modulation with the mitochondria uncouplers SR4 and niclosamide prevents proliferation and growth of treatment-naïve and vemurafenib-resistant melanomas
Source: Oncotarget. 2018 Dec 11;9(97):36945–65. doi: 10.18632/oncotarget.26421 (PMC6319337; doi:10.18632/oncotarget.26421)
Supplement: Supplementary file 1 [file oncotarget-09-36945-s001.pdf]

Bioenergetic modulation with the mitochondria uncouplers SR4 and niclosamide prevents proliferation and growth of treatment-naïve and vemurafenib-resistant melanomas

SUPPLEMENTARY MATERIALS

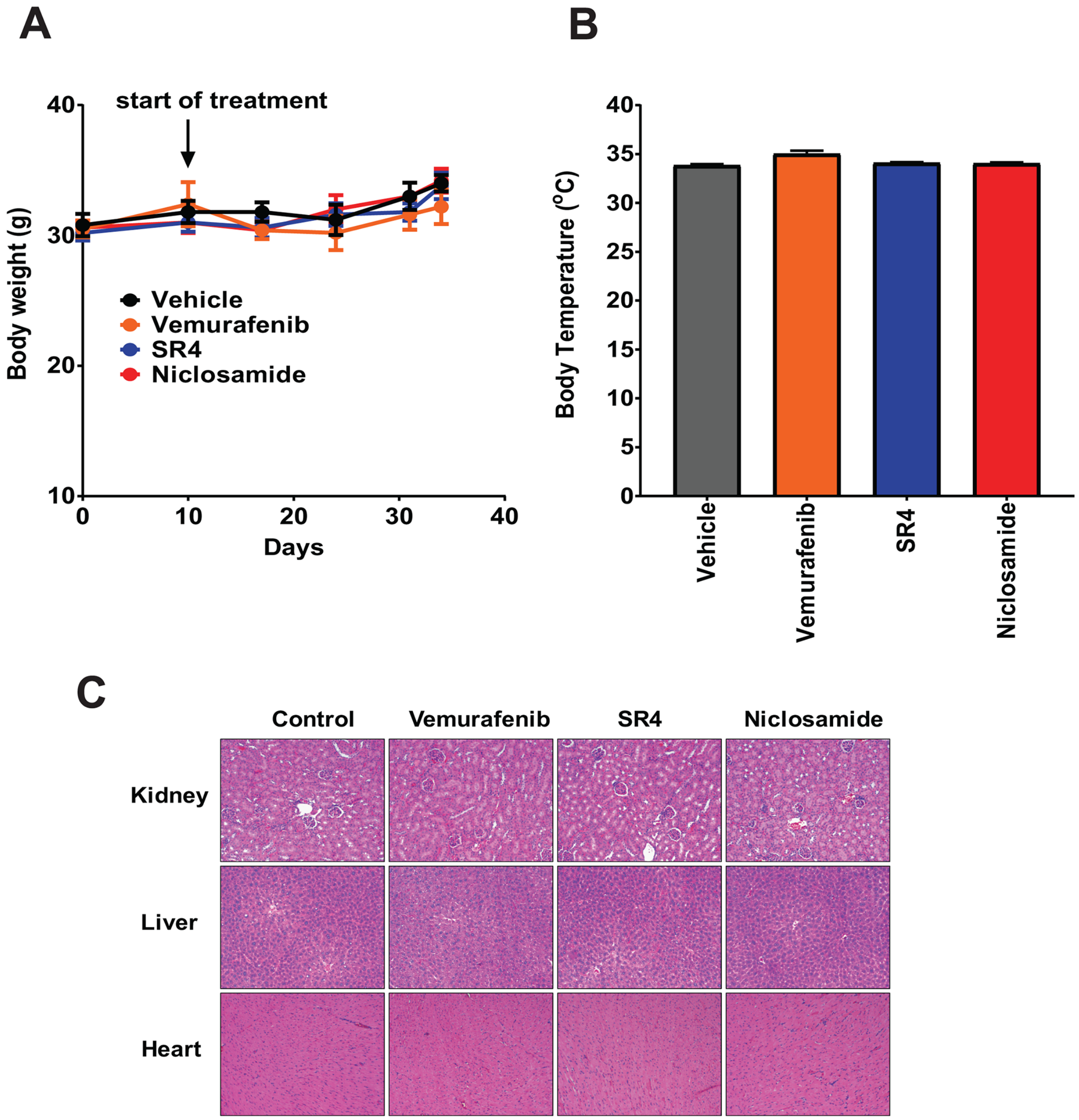

Supplementary Figure 1: Effects of SR4, niclosamide and vemurafenib on body weight, body temperature and key organs of nude mice bearing the A375 xenograft. **(A)** Body weights of mice during treatment of test compounds each administered orally at 10 mg/kg/day. Data are means ± SEM (n=6). **(B)** Body temperature of mice after 4 hr treatment of test compounds (10 mg/kg/day, p.o). Data are means ± SEM (n=6). **(C)** Representative H&E staining of kidney, heart and liver sections from xenograft mice in each treatment group.

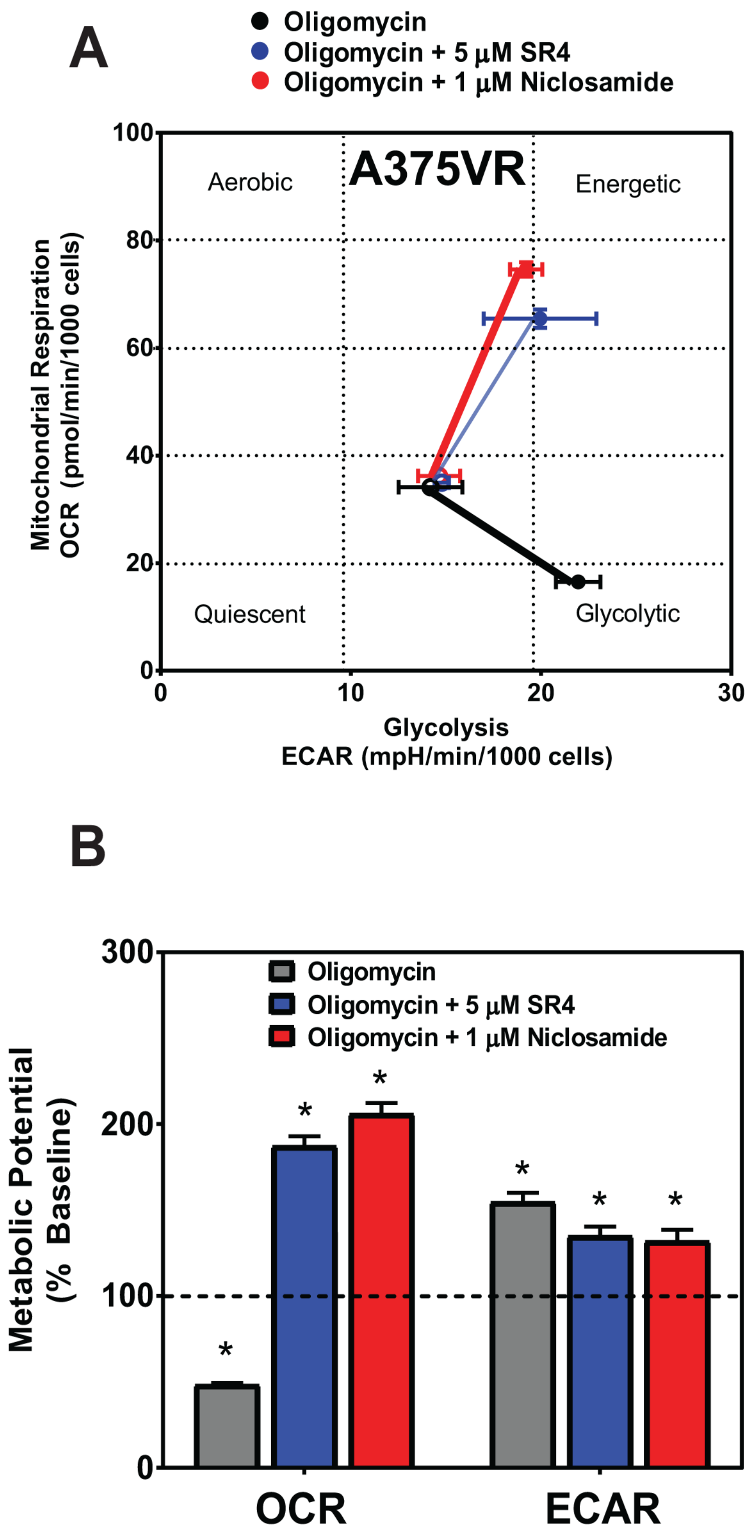

Supplementary Figure 2: SR4 and niclosamide increase metabolic potential in vemurafenib-resistant melanoma. (A) Metabolic phenogram analysis of OCR and ECAR by Seahorse Cell Energy Phenotype test. A375VR cells were treated with 1 μM oligomycin with or without SR4 (5 μM) or niclosamide (1 μM) and the change in metabolic phenotype was assessed after 1 h treatment. **(B)** Metabolic OCR and ECAR potential of A375VR cells treated with oligomycin alone or oligomycin + SR4 or niclosamide as determined from the Cell Energy Phenotype test. Data in all three figures are means from 6-8 wells ± SEM, and are representative rates of 2 separate experiments. $^{*}P < 0.05$ vs. baseline levels.

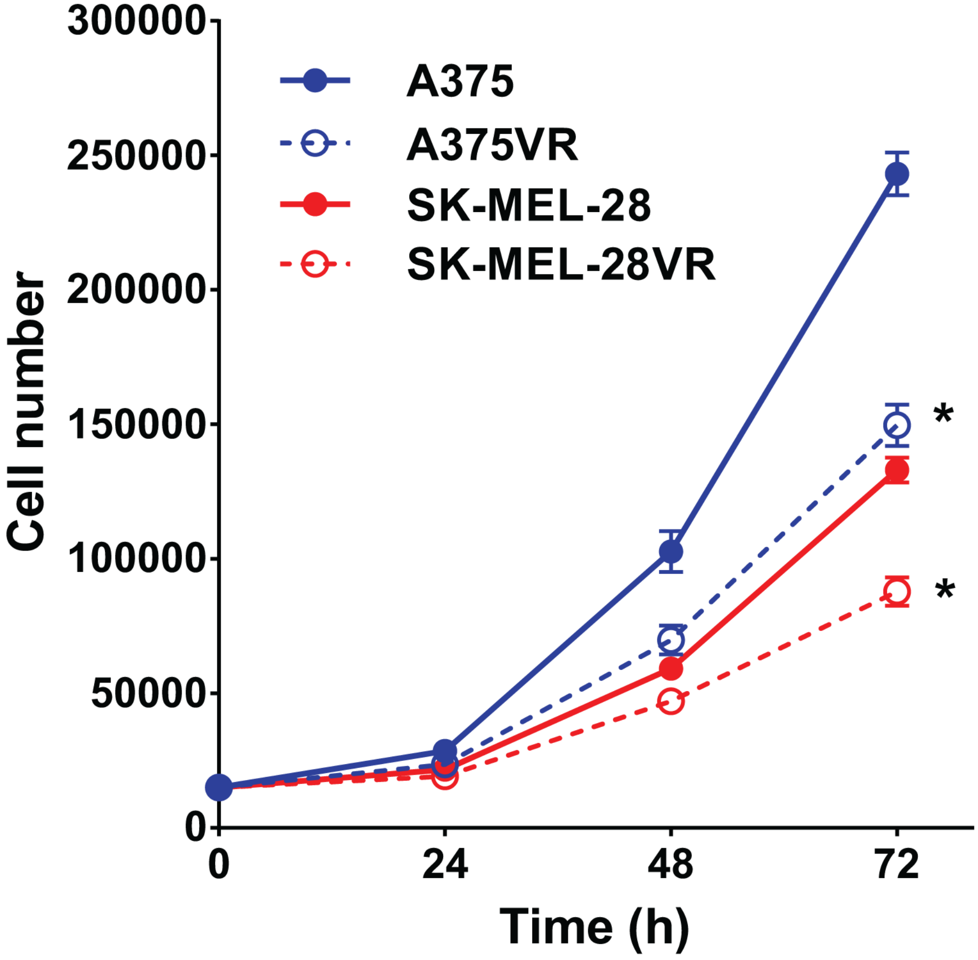

Supplementary Figure 3: Cell growth comparison between parental and vemurafenib-resistant melanoma cell lines. Each cell line was plated at 15,000 cells/well at time=0. After 24, 48, and 72 h of incubation, the cell number in each well was measured using an automated cell counter (Coulter Z1, Beckman Coulter, Brea, CA). Data are means ± SEM from three replicates per time point. $^{*}P < 0.05$ vs. parental cell.

Supplementary Table 1: Comparison of growth inhibition (GI_{50}) data of niclosamide and SR4 from the NCI-DTP60 panel screening. Highlighted in *yellow* are the melanoma cell lines. In all cell panels, niclosamide had lower GI_{50} than SR4, except in the two colon cancer lines highlighted in *green*.

See Supplementary File 1
